# Presence of Micro- and Nanoplastics Affects Degradation of Chlorinated Solvents

**DOI:** 10.3390/toxics13080656

**Published:** 2025-07-31

**Authors:** Fadime Kara Murdoch, Yanchen Sun, Mark E. Fuller, Larry Mullins, Amy Hill, Jacob Lilly, John Wilson, Frank E. Löffler, Katarzyna H. Kucharzyk

**Affiliations:** 1 Battelle Memorial Institute, 505 King Ave, Columbus, OH 43201, USA; karamurdoch@battelle.org (F.K.M.); mullinsl@battelle.org (L.M.); hilla@battelle.org (A.H.); lillyj@battelle.org (J.L.); 2 Department of Civil and Environmental Engineering, University of Tennessee, Knoxville, TN 37996, USA; yanchen.sun@whoi.edu (Y.S.); frank.loeffler@utk.edu (F.E.L.); 3 Department of Biochemistry & Cellular and Molecular Biology, University of Tennessee, Knoxville, TN 37996, USA; 4 Department of Biosystems Engineering, University of Tennessee, Knoxville, TN 37996, USA; 5 Aptim Federal Services, LLC, 17 Princess Road, Lawrenceville, NJ 08648, USA; mark.fuller@aptim.com; 6 Scissortail Environmental Solutions, LLC, 2013 Foster Drive, Ada, OK 74820, USA; john@scissortailenv.com; 7 Biosciences Division, Oak Ridge National Laboratory, Oak Ridge, TN 37831, USA

**Keywords:** microplastics, nanoplastics, chlorinated solvents, explosives, *Dehalococcoides mccartyi*, *cis*-DCE stall, polystyrene, polyamide 6, RDX, TCE

## Abstract

Microplastics (MPs) and nanoplastics (NPs) can affect microbial abundance and activity, likely by damaging cell membrane components. While their effects on anaerobic digestion are known, less is understood about their impact on microbes involved in contaminant bioremediation. Chlorinated volatile organic contaminants (CVOCs) such as tetrachloroethene (PCE) and explosives like hexahydro-1,3,5-trinitro-1,3,5-triazine (RDX) are common in the environment, and their bioremediation is a promising cleanup strategy. This study examined how polystyrene (PS) and polyamide 6 (PA6) MPs and NPs influence CVOC and RDX biodegradation. PS particles did not inhibit the CVOC-degrading community SDC-9, but PA6 MPs impaired the reductive dechlorination of trichloroethene (TCE) to cis-1,2-dichloroethene (*cis*-DCE), causing a “*cis*-DCE stall” with no further conversion to vinyl chloride (VC) or ethene. Only 45% of TCE was dechlorinated to *cis*-DCE, and *Dehalococcoides mccartyi* abundance dropped 1000-fold in 35 days with PA6 MPs. In contrast, neither PA6 nor PS MPs and NPs affected RDX biotransformation. These results highlight the significant impact of PA6 MPs on CVOC biodegradation and the need to consider plastic pollution in environmental management.

## 1. Introduction

Groundwater contamination by various anthropogenic organic compounds is a widespread problem with numerous chemicals persisting in the subsurface [1]. Many organic contaminants are biodegradable under oxic and/or anoxic conditions, with biological systems facilitating their degradation reactions. Chlorinated volatile organic contaminants (CVOCs) and explosives are widely distributed environmental contaminants present both in sediments and groundwater [2]. The most common strategy for biodegradation of CVOCs, such as tetrachloroethene (PCE), is anaerobic microbial reductive dechlorination to trichloroethene (TCE), dichloroethenes (DCEs), vinyl chloride (VC) and ethene. Although several microbial groups are capable of reductive dechlorination of PCE and TCE to *cis*-DCE, only some *Dehalococcoides* (*Dhc*) strains, such as strains VC (*cis*-DCE, 1,1-DCE and VC, to ethene) and BAV1 (all DCE isomers and VC to ethene) [3,4], and *Dehalogenimonas etheniformans* can dechlorinate DCEs and VC to benign ethene [5]. Hexahydro-1,3,5-trinitro-1,3,5-triazine (RDX) biodegradation has been observed under oxic [6,7] and anoxic conditions via sequential reduction of the nitro groups to achieve mono-, di- and trinitro derivatives (MNX, DNX and TNX, respectively) [8,9]. In *Gordonia* sp. strain KTR9, the enzymes responsible for RDX degradation under aerobic conditions are the cytochrome P450 monooxygenase XplA and flavodoxin reductase XplB [10]. *Pseudomonas fluorescens* I-C degrades RDX primarily under anoxic or low-oxygen conditions utilizing the flavoprotein oxidoreductase XenB [11]. Several factors may affect the biodegradation of organohalide contaminants, for example, the presence of toxic compounds, lack of nutrients, accumulation of by-products and environmental parameters such as pH, temperature, oxygen, etc. Other factors, such as the presence of micro- and nanoparticulate plastic particles, have been less investigated, and contradictory results have been reported [12,13,14].

Synthetic plastics are extensively used in daily life and enter the environment, where they form decreasingly smaller particles that accumulate in soils, sediments and aqueous systems. The study of plastic contamination is a relatively new area of investigation with several challenging questions remaining in relation to their impacts on the natural environment, biotic processes and microbial life [15,16]. To date, plastics research focuses on surface waters (oceans and rivers), with the effects of plastic presence and implications on aquifer contamination receiving increasing attention. Plastic particles under 5 mm in size are considered microplastics [17], while nanoplastics are in the size range of 1–1000 nm and exhibit colloidal behavior [18]. Their chemical and physical properties allow for adsorption to hydrophobic compounds, and it is possible that they have high potential to adsorb and desorb toxic contaminants such as polycyclic aromatic hydrocarbons (PAHs), polycyclic chlorinated biphenyls (PCBs) and metals [19,20,21]. Many studies show that microplastics (MPs) and nanoplastics (NPs) can inhibit various biological reactions such as nitrification, denitrification and phosphorus removal [22,23,24]. Microplastics are known to reduce the abundance of specific microorganisms [24]. For example, nitrogenase reductase (*nifH*) and ammonium monooxygenase (*amoA*) [25] genes were detected with significantly fewer gene copies when assessing nitrogen assimilation processes by microbial consortia in the presence of MPs. An inhibitory effect of polyethylene MPs on the reductive dechlorination of Aroclor 1260 [12] and the growth inhibition of *Escherichia coli* (*E. coli*) in the presence of 1.7 and 2.3 µm PS MPs have been reported [26]. Several studies have indicated that microplastic particles can provide a surface for attachment of microorganisms [27] and further impact microbial growth [26]. Some studies have shown that nanoplastics can interact with microbial cell membranes and cause elevated oxidative stress, which may lead to gene mutations, growth inhibition and decreased enzyme activity [13,28,29,30]. Recently, several studies highlighted the potential adverse effects of certain types of plastics on anaerobic digestion [31,32]. Liu et al. [33] reported that brief exposure to individual microplastics and nanoplastics (such as polyethylene, polyvinyl chloride, polystyrene and polylactic acid) at a low concentration (10 particles per gram of dry sludge) modestly increased methanogenesis by 2.1% to 9.0%. However, at higher concentrations (30 to 200 particles per gram of dry sludge), methanogenesis was reduced by 15.2% to 30.1%. Theoretically, the greater the abundance of plastic particles, the stronger the inhibition. Recent studies also revealed that microbial cells exposed to polystyrene (PS) nanoparticles undergo bioflocculation due to the alteration of their exopolysaccharide fractions [34]. The adsorption of microplastics to biomolecules on the cell surface may result in the reduction or inactivation of enzyme activity, accordingly affecting metabolic performance [35].

As many studies to date have explored the implications of widely distributed polyethylene or polyether terephthalate polymers on microbial life, this study focused on less commonly studied plastic polymers. The objective of this study was to determine the effects of less prevalent but still fairly abundant MPs and NPs, specifically PS and PA6 (also known as Nylon 6), on microbial degradation processes of chlorinated ethenes and RDX. These types of plastic particles were selected due to being commonly found plastic particles in surface waters, sediments and soils [36,37]. Also, given their unique physiochemical characteristics and widespread application, understanding their distinct interactions with environmental processes, such as microbial activity, is crucial for a comprehensive assessment of their effect(s) on microbial processes like biodegradation of contaminants. Therefore, different sizes and concentrations of PS and PA6 were evaluated in this study. Specifically, the commercially available microbial consortium SDC-9 (used to enhance reductive dechlorination of chlorinated ethenes) and pure cultures of RDX degraders—*Gordonia* sp. strain KTR9 and *Pseudomonas fluorescens* strain I-C—were used to examine the effects of plastic particle type and size on degradation efficiency.

## 2. Materials and Methods

### 2.1. Materials

Polybead^®^ Polystyrene microspheres with sizes of 10.0 μm (Catalog No. 17136-5) and 50.0 nm (Catalog No. 08691-10) were purchased from Polysciences Inc. (Warrington, PA, USA) as a 2.5% solid (*w*/*v*) (≈25 mg mL^−1^) aqueous suspension. The reported number of particles per mL was 4.55 × 10^7^ and 3.64 × 10^14^ for PS MPs (with 10 μm mean diameter) and PS NPs (with 50 nm mean diameter), respectively.

PA6 spheroidal powder with a 20 µm average diameter (Catalog No. AM30601, 780-705-41) was purchased from Goodfellow Advanced Materials (Pittsburg, PA, USA). The number of particles per gram was calculated as 5.62 × 10^7^. PA6 NPs were synthesized using a protocol adapted from Crespy and Landfester (2007) [38]. The synthesized PA6 was analyzed using dynamic light scattering to determine the particle size (Malvern Zetasizer Nano ZS, Westborough, MA, USA). The amount was determined gravimetrically after evaporation at 100 °C. The average size was determined as ~336 nm (Figure S1). The number of PA6 NP particles per mL was calculated as 4.42 × 10^9^.

TCE, *cis*-DCE, VC (each at ≥99.5%), ethene (≥99.9%), Vitamin B_12_ (≥98%), RDX (≥98%) and sodium lactate (≥98%) were purchased from Sigma-Aldrich (St. Louis, MO, USA).

### 2.2. Preparation of MP and NP Suspension Solutions

A plastic concentration of 5 × 10^3^ particles per mL (8.9 mg L^−1^ for PA6, 2.7 mg L^−1^ for PS) was selected as environmentally relevant for testing [39]. The effects of 5 × 10^5^ particles per mL (889.7 mg L^−1^ for PA6 and 274.7 mg L^−1^ for PS) were also investigated due to the potential continuous increase in the presence of PS and PA6 MPs and NPs in soils and sediments and also because the actual environmental concentrations of these particles are likely vastly underestimated due to analytical challenges in detecting and quantifying micro- and nanoplastic particles in complex environmental matrices. To prepare sterile stock suspension solutions, MPs and NPs were dispersed in 100 mL of sterile Milli-Q water by sonication for 60 min (room temperature, 40 kHz) [40]. The size of MP and NP particles in suspension was determined using fluorescence-activated cell sorting flow cytometry (Accuri C6 Plus Flow Cytometer, San Jose, CA, USA) and dynamic light scattering (Malvern Zetasizer Nano ZS, Westborough, MA, USA). The PS calibration standard mix was prepared with particles with diameters of 6 µm, 10 µm and 15 µm (Invitrogen, F13838, Carlsbad, CA, USA) and 30 µm (Sigma Aldrich, 84135, St. Louis, MA, USA).

### 2.3. SDC-9 Culture and Growth Conditions

The SDC-9 microbial culture [41] was an in-kind donation from APTIM Inc. (Baton Rouge, LA, USA) and was grown in 160 mL serum bottles containing 100 mL of bicarbonate-buffered (30 mM, pH 7.3) mineral salt medium with 60 mL headspace filled with CO_2_/N_2_ (20/80, *vol*/*vol*), as described previously [42]. The medium was amended with Wolin vitamin mix containing B_12_ to achieve a final concentration of 25 μg L^−1^. Each experimental vessel received 10 mM lactate as an electron donor and approximately 55 μmol TCE as an electron acceptor. Each culture was inoculated (0.1%, *v*/*v*) from an active TCE-dechlorinating SDC-9 culture to a final *Dehalococcoides mccartyi* (*Dhc*) cell abundance of 1 × 10^8^ cells L^−1^, as determined by quantitative PCR. The microbial composition of the SDC-9 culture was known and had been previously reported by Kucharzyk et al. [43]; hence, the qPCR primers previously designed and reported for *Dhc* were used in this study [44].

### 2.4. RDX Cultures and Growth Conditions

*Gordonia* sp. strain KTR9 [45] and *Pseudomonas fluorescens* strain I-C [11] (KTR9 and I-C, respectively) were propagated in sterile basal salt medium (BSM) as previously described [46] with modifications. Briefly, experiments were performed in sterile 40 mL glass vials with Teflon-lined caps and approximately 23 mL of appropriate medium (e.g., BSM, 0.1 mM fructose, RDX for I-C; BSM without ammonium (BSM-N), 0.1 mM fructose, RDX for KTR9). I-C experiments were performed under a nitrogen headspace to facilitate anoxic RDX degradation. Inoculum cultures were harvested, washed with BSM-N and then added to achieve an initial cell density of approximately 1 × 10^9^ cells mL^−1^. The optical density of cultures was recorded at 600 nm (OD_600_) using a Spectronic Genesys 2 UV/Vis Spectrophotometer (Thermo Fischer Scientific, Waltham, MA, USA) during the experiment to detect any additional growth attributed to the consumption of RDX.

### 2.5. Adsorption/Desorption Experiment Setup

The adsorption/desorption of TCE and RDX to MPs and NPs was tested. For CVOCs and ethene, initially, 5 × 10^5^ PA6 microplastic particles per mL were suspended in 100 mL mineral salt medium in 160 mL glass serum bottles and approximately 55 μmol TCE, 60 μmol *cis*-DCE, 70 μmol VC and 75 μmol ethene per 160 mL bottle was added separately. The bottles were closed with black butyl rubber stoppers and incubated upright without agitation at 30 °C in the dark, mimicking the setup of the CVOC degradation experiments. The bottles with CVOCs and ethene in the presence of MPs and NPs were incubated for 10 days to provide enough time for adsorption/desorption under static conditions. An identical setup was repeated with 5 × 10^5^ PS microplastic particles per mL, but the sets were monitored for 5 days based on the results from the adsorption/desorption experiments with PA6 MPs and NPs. Adsorption of VC, ethene and cis-DCE was not observed in the presence of 5 × 10^5^ PA6 and PS particles/mL and the adsorption/desorption experiments were only performed with TCE in the presence of 5 × 10^3^ and 5 × 10^5^ PS and PA6 NPs and MPs. All experiments were performed in duplicate, and bottles without MPs and NPs served as controls. Headspace samples (0.1 mL) were periodically withdrawn, and TCE, *cis*-DCE, VC and ethene concentrations were quantified by GC with an FID detector as previously described [47].

For RDX adsorption/desorption experiments, 42 µmol L^−1^ RDX was added, and the bottles were agitated for 72 h with low and high particle concentrations of each micro- and nanoplastic polymer. For control sets, the bottles were prepared with 42 µmol L^−1^ RDX and agitated without the addition of MPs and NPs. The bottles were shaken at 200 rpm at 22 °C, mimicking the conditions of the RDX degradation experiments. All experiments were performed in duplicate. The concentration of the RDX was determined using high-pressure liquid chromatography (HPLC) with previously described methods [48].

### 2.6. Impact of MPs and NPs on Reductive Dechlorination

To assess the effect of MPs and NPs on reductive dechlorination performance, MP or NP suspensions were added directly to the experimental vessels on Day 3 to achieve final particle counts of 5 × 10^3^ and 5 × 10^5^ per mL. The experiments were performed in triplicate, and culture bottles without MP/NPs and without SDC-9 cells served as biotic and abiotic controls, respectively (Set 1). Culture bottles were incubated without agitation at 30 °C in the dark with the stoppers facing up. TCE and dechlorination products were monitored periodically. The microcosm study in the presence of PS and PA6 at 5.0 × 10^5^ particles mL^−1^ was repeated (Set 2) to increase the statistical power of the measurements and to allow for increased credibility of any critical data generated from the study.

### 2.7. Effect of MP and NP Presence on RDX Degradation

Two sets of experiments were set up to test the effects of plastics on the degradation of RDX. Experimental samples consisting of strain KTR9 and strain I-C were cultivated in their respective media with low and high particle concentrations of each micro- and nanoplastic polymer spiked immediately after culture inoculation. Vials were placed sideways and shaken at 200 rpm at 22 °C. Controls were also included to account for abiotic losses (e.g., sorption).

To monitor RDX transformations, samples were periodically collected and centrifuged at 14,000× *g* for 3 min to remove cells and plastic particles. The supernatant was diluted 1:1 with methanol and analyzed using HPLC. Approximately 60 mL of sample aliquots were collected for proteomics at the end of each experiment. Samples were frozen at −80 °C until further analysis.

### 2.8. Analytical and Statistical Procedures

The concentrations of TCE and products of reductive dechlorination (i.e., *cis*-1,2-DCE, VC and ethene) were measured by manual headspace injections (0.1 mL) into an Agilent 7890A gas chromatograph (GC) (Santa Clara, CA, USA) equipped with a DB-624 column (60 m length, 0.32 mm, 1.8 mm film thickness) and a flame ionization detector (FID) [47]. The GC inlet was maintained at 200 °C; the GC oven temperature was kept at 60 °C for 2 min followed by an increase to 200 °C at a ramping rate of 25 °C min^−1^; and the FID detector was operated at 300 °C. The concentration of RDX was determined using HPLC with previously described methods [48]. A correlation between analytical data and CVOC and RDX concentrations was developed and plotted using SigmaPlot (San Jose, CA, USA). A linear regression of the data was performed, and R^2^ values were then calculated.

To determine significant differences between the treatment (in the presence of MPs/NPs) and control group means, a one-way analysis of variance (ANOVA) was conducted. When overall significant differences were identified, Tukey’s post hoc test was subsequently applied to perform pairwise comparisons between specific group means. A significance level of *p* < 0.05 was used for all statistical tests. All analyses were performed with SPSS v22 (IBM Corporation, Armonk, NY, USA).

### 2.9. Quantitative Proteomics (qProt)

Proteins were extracted, spiked with a cocktail of 250 fmol/µL isotopically labeled peptides, as previously described [49,50], and digested and purified using previously developed methods [51]. Bovine serum albumin was used to monitor digestion efficiency. An indexed retention time (iRT) peptide cocktail (Sciex) was used to monitor instrument performance. Following digest downstream processing, 10 µL of sample was injected into the Xevo triple quadrupole mass spectrometer (Waters, Milford, MA, USA).

Mass spectrometric parameters for the analysis of target proteins on the Xevo TQ-XS triple quadrupole mass spectrometer were optimized for *Dhc* biomarker peptides developed previously by our team [51]. Isotopically labeled peptides were injected via syringe infusion, and precursor-to-product ion transitions were identified and optimized using the IntelliStart feature in MassLynx v4.2 software (Waters, Milford, MA, USA). All determined parameters for the isotopically labeled peptides were then used for their corresponding native peptides. An unscheduled selected reaction monitoring method was created using the top two most abundant transitions for each target peptide. Sample MS/MS data were acquired using an Acquity M-class liquid chromatograph system (Waters, Milford, MA, USA) directly connected to a Xevo TQ-XS mass spectrometer [50]. Detailed description of parameters is provided in the Supplementary Information (Table S1) Section.

### 2.10. DNA Extraction and Quantitative PCR

Samples were collected and washed with phosphate-buffered saline buffer, and DNA was isolated from cell pellets with DNeasy PowerLyzer PowerSoil Kit (Qiagen, Germantown, MD, USA) according to the manufacturer’s instructions with bead-beating (OMNI Bead Ruptor, 5 m/s for 3 min) (OMNI International, Kennesaw, GA, USA) to enhance cell lysis. DNA was eluted into nuclease-free water, and the concentration was determined with a Qubit fluorometer (Invitrogen, Carlsbad, CA, USA) using a double-stranded DNA Broad-Range assay kit according to the manufacturer’s instructions. DNA was stored at −80 °C until analysis.

The abundance of *Dhc* 16S rRNA genes, TCE reductive dehalogenase (RDase) (*tceA*, TCE to VC), VC RDase (*vcrA*, cis-DCE/VC to ethene) and organohalide respiration molybdoenzyme (*omeA*) genes was determined using previously reported TaqMan quantitative PCR (qPCR) assays [44,49,51].

qPCR was performed using the QuantStudio 12K Flex Real-Time PCR System (Applied Biosystems [ABI], Carlsbad, CA, USA). Every 10 µL reaction consisted of 5 μL of 2× Taqman Universal PCR Master Mix (ABI), 2 μL of diluted (1:10) DNA template and forward and reverse primers and probes at final concentrations of 300 nM each. Reactions were initially held for 2 min at 50 °C and 10 min at 95 °C following 40 cycles of denaturation at 95 °C for 15 s and annealing and extension at 60 °C for 1 min. The qPCR results were analyzed using the QuantStudio 12K Flex Real-Time PCR System Software (ABI, Carlsbad, CA, USA). Calibration curves used serial 10-fold dilutions of synthetic linear DNA fragments (1500 bp) with partial gene fragments of *tceA*, *vcrA*, *omeA* and 16S rRNA genes comprising the primer-binding sites and spanning a concentration range of approximately 10^1^ to 10^8^ target gene copies/µL. The linear DNA fragment was synthesized by Invitrogen (GeneArt Strings DNA Fragments; Invitrogen, Carlsbad, CA, USA).

## 3. Results and Discussion

### 3.1. Sorption/Desorption Studies

**Figure 1:**
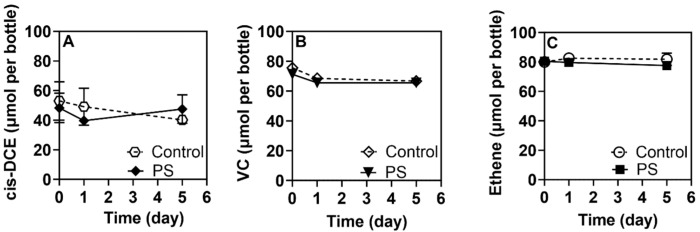
Adsorption behavior of *cis*-DCE, VC and ethene in the presence of 5 × 10^5^ PS microplastics particles per mL. (**A**) *cis*-DCE, (**B**) VC and (**C**) ethene concentrations during the 5-day adsorption study. Each time point is plotted as an average of duplicate measurements. Error bars are calculated as the standard error from duplicate measurements.

**Figure 2:**
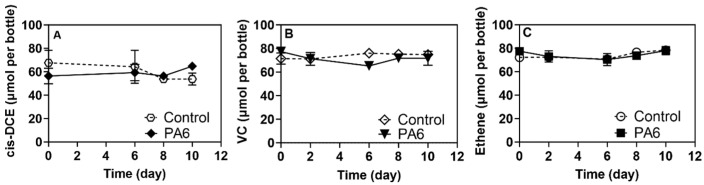
Adsorption behavior of cis-DCE, VC and ethene in the presence of 5 × 10^5^ PA6 microplastics particles per mL. (**A**) *cis-*DCE, (**B**) VC and (**C**) ethene concentrations during the 10-day adsorption study. Each time point is plotted as an average of duplicate measurements. Error bars are calculated as the standard error from duplicate measurements.

**Figure 3:**
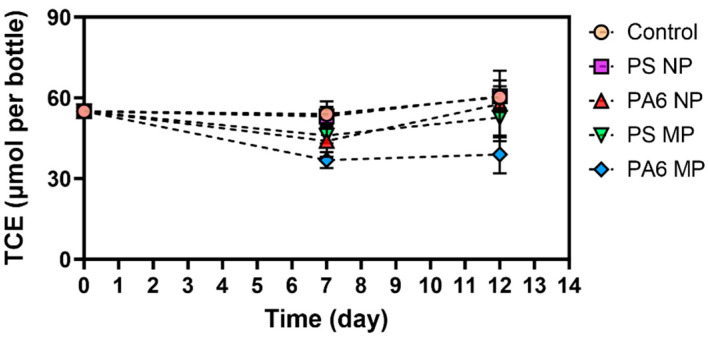
Adsorption behavior of TCE in the presence of 5 × 10^5^ PA6 and PS microplastic and nanoplastic particles per mL during the 12-day adsorption study. Each time point is plotted as an average of duplicate measurements. Error bars are calculated as the standard error from duplicate measurements.

To rule out any abiotic interactions of the MPs and NPs with target contaminants, which could create false positive effects, the sorption of CVOCs and RDX was evaluated. The incubation of MPs and NPs with RDX showed negligible contaminant-particle affinity regardless of the polymer particle size (Figure S2). Data from experiments conducted with cis-DCE, VC and ethene did not indicate any sorption to PA6 and PS (Figure 1 and Figure 2). However, TCE showed significant sorption, with nearly 31 ± 2.68% of the starting TCE concentration associated with the PA6 microplastic particles when 5 × 10^5^ particles mL^−1^ were added to the medium (Figure 3). This high affinity is not surprising, as TCE is more hydrophobic than cis-DCE and VC [52]. Interestingly, PS NPs and MPs at the studied level did not show any statistically important adsorption of TCE. This could be attributed to the type of plastic polymer and its properties, which determine sorption/desorption behavior [53,54].

The PA6 NPs, when spiked at 5 × 10^5^ particles mL^−1^, did not exhibit a noteworthy adsorption of TCE. Thus, it is possible that the sorption of the chloroethenes to PA6 MPs, which are larger-sized plastic particles, may play an important role in decreasing the aqueous concentration of organic pollutants. Sorption affects the stored mass of a contaminants, the effective rate of diffusion and, therefore, the mass transfer of contaminants between the adsorbed phases on the matrix and the mobile aqueous phase [55]. In the published literature, a positive correlation was shown between the number of plastic particles and the concentrations of polychlorinated biphenyls (PCBs), another group of chlorinated contaminants. Thus, it is important to investigate the simultaneous sorption of chlorinated solvents and energetic compounds to microplastics to characterize their behavior. Previous research has shown that strong sorption of hydrophobic organic chemicals to carbon-based particles is caused by a combination of hydrophobic interactions and π–π interactions at the aromatic surface and therefore is highly dependent on specific surface area [56]. A recent study showed the presence of PAH in virgin PS pellets, with higher sorption of PAH to PS MPs than to nonaromatic polyethylene, polyvinyl chloride and polypropylene microplastics [56]. The higher affinity of TCE for PA6 than for other chlorinated compounds may indicate a differential binding mechanism related to the nature of the chemical, partition of the polymer and pore filling mechanism on the surface of the plastic particles. The specific chemical structure of PA6 (e.g., amide groups that can form hydrogen bonds) may make them effective sorbents for various organic compounds. When TCE binds strongly to the PA6 microplastic surface, it is effectively sequestered from the aqueous phase. Since *Dehalococcoides* and other dehalogenating bacteria typically access their electron acceptors (like TCE) in dissolved form, this reduction in the freely available aqueous concentration of TCE directly limits their metabolic access to the substrate. Consequently, the rate of reductive dehalogenation would be diminished, not necessarily due to a direct toxic effect of the plastic particles on the microbes but rather an indirect effect stemming from substrate limitation. However, the relationship between the structure of the polymer and sorption effects is still unclear.

### 3.2. Presence of Plastic Particles Inhibits CVOC Degradation

**Figure 4:**
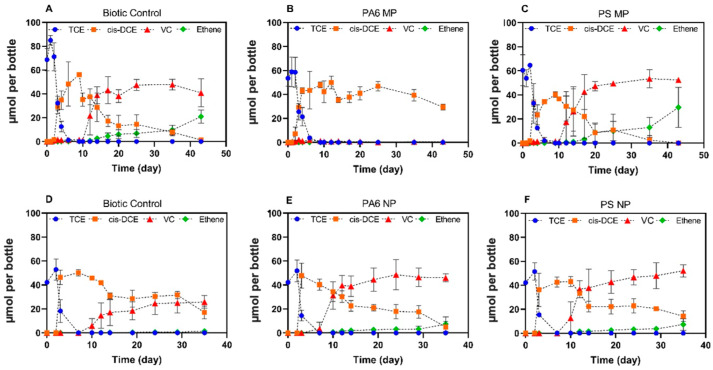
Effect of 5.0 × 10^5^ particles per mL of MPs and NPs on reductive dechlorination of TCE. (**A**) Biotic control MP, SDC-9 dechlorinating culture without any plastic addition; (**B**) addition of 5 × 10^5^ particles PA6 (20 µm) on Day 3; (**C**) addition of 5 × 10^5^ particles PS (10 µm) on Day 3; (**D**) biotic control NP, SDC-9 dechlorinating culture without addition of 5 × 10^5^ particles PA6 (336 nm) and PS (10 nm) nanoplastics; (**E**) addition of 5 × 10^5^ particles PA6 (336 nm) on Day 3; and (**F**) addition of 5 × 10^5^ particles PS (10 nm) on Day 3. Vertical error bars denote the standard deviation.

Microbially driven dechlorination of CVOCs in contaminated aquifers may be inhibited by variety of factors [57,58], including the presence of plastic particles. No significant inhibition (*p* > 0.05) was observed for the reductive dechlorination activity of SDC-9 culture spiked with PS and PA6 MPs and NPs at 5.0 × 10^3^ particles mL^−1^. However, the reductive dechlorination of TCE by SDC-9 culture in the presence of PA6 MPs at 5.0 × 10^5^ particles mL^−1^ (added on Day 3) for the Set 1 experiment revealed a detrimental effect of PA6 MPs on the reductive dechlorination of TCE (*p* < 0.05) by SDC-9 dechlorinating culture (Figure 4). The intention of adding MPs/NPs on Day 3 was to introduce them to the dechlorinating microbial culture during its early development.

In the absence of PA6 MPs, SDC-9 culture completely dechlorinated TCE to *cis*-DCE over a 43-day incubation period with the formation of VC (Figure 4A,D). The degradation of TCE resulted in the formation of *cis*-DCE followed by its rapid conversion to VC. The formation of ethene from VC, the final reductive dechlorination step, was incomplete after 6 weeks. When the TCE was amended, SDC-9 biotic control cultures converted 68.8 ± 9.4 µmol of TCE completely to *cis*-DCE (60.0 ± 1.8 µmol) in 10 days and then to VC (40.9 ± 11.8 µmol) and ethene (21.0 ± 5.5 µmol) after a 43-day incubation period, but the cultures were terminated before the complete conversion of VC to ethene (Figure 4A). In the microcosms where PA6 MPs and NPs were spiked (Figure 4B,E) in the culture, the data for TCE in the presence of PA6 MPs demonstrated a *cis*-DCE stall with no ethene formation. Only 45% of the initial TCE was dechlorinated to *cis-*DCE (29.4 ± 2.5 µmol), with 1% formation of VC (0.5 ± 0.9 µmol) in the presence of PA6 MPs during a 43-day incubation period. TCE was degraded at a rate of 179.5 ± 35.3 μmol L^−1^ day^−1^ in the biotic control cultures, which was greater than in cultures that received PA6 MPs (119.6 ± 23.4 μmol L^−1^ day^−1^) and PS MPs (126.5 ± 31.9 μmol L^−1^ day^−1^). Although *cis-*DCE was degraded at similar rates in the biotic control (12.6 ± 2.9 μmol L^−1^ day^−1^) and PS MPs (14.6 ± 2.4 μmol L^−1^ day^−1^) cultures, PA6 MP cultures exhibited a degradation rate of *cis*-DCE of 3.4 ± 1.6 μmol L^−1^ day^−1^. Electron donor limitation cannot be the reason for the *cis*-DCE stall because H_2_ was provided in excess amount based on the electron demand to complete the conversion of TCE to ethene. In contrast to the complete reductive dechlorination of TCE to ethene in biotic controls without plastic particles, negligible amounts (0.5 ± 0.9 μmol) of VC and no ethene were formed in cultures with PA MPs, indicating that the cDCE-to-VC step was susceptible to PA6 inhibition. An independent experiment corroborated these findings (Figure S3). In contrast, no inhibitory effects on reductive dechlorination were observed for PS MP cultures (Figure 4C).

The addition of 5 × 10^5^ particles mL^−1^ of nano-scale PS and PA6 did not cause measurable inhibitory effects on the SDC-9 culture. Cultures without PS NPs (biotic control, Figure 4D) completely dechlorinated the initial amount of 42.2 μmol of TCE to *cis*-DCE within 7 days, with formation of VC (25.8 ± 5.2 μmol) and ethene (1.4 ± 0.9 μmol) over a 35-day incubation duration. In the same period, SDC-9 cultures demonstrated dechlorination of TCE to a mixture of 46.1 ± 3.4 μmol VC and 7.4 ± 6.0 μmol ethene in the PA6 NP cultures and 52.1 ± 5.1 μmol VC and 7.3 ± 4.8 μmol ethene in the PS NP cultures. In contrast to the *cis*-DCE degradation (9.60 ± 2.1 μmol L^−1^ day^−1^) and ethene production (0.4 ± 0.06 μmol L^−1^ day^−1^) rates for biotic controls with NPs (Figure 4D), *cis-*DCE degradation and ethene formation rates of 10.7 ± 1.5 μmol L^−1^ day^−1^ and 2.1 ± 0.4 μmol L^−1^ day^−1^, respectively, for PA6 NPs cultures and 9.5 ± 2.2 μmol L^−1^ day^−1^ and 2.3 ± 0.4 μmol L^−1^ day^−1^, respectively. for PS NPs cultures were observed. The results indicated that PS NPs at a concentration of 5 × 10^5^ particles mL^−1^ did not exhibit any negative effects on the reductive dechlorination of TCE to ethene. The observed lack of detrimental effect of polystyrene (PS) particles on microbial activity could be attributed to several factors. Primarily, the inherent resilience of the SDC-9 microbial community may play a significant role, as different microbial species and consortia exhibit varying sensitivities to stressors. It is plausible that the specific composition of the SDC-9 culture is more tolerant to the presence of PS particles than communities assessed in other studies. Furthermore, the surface chemistry of the PS particles themselves could contribute to this outcome. Rather than inducing stress, the specific surface properties of the PS particles used in this study might be more benign or even provide a favorable surface for microbial attachment and colonization, potentially mitigating any negative impacts on overall community activity.

Overall, these experiments show that the presence of MPs at a high abundance relevant to field conditions (average 1000 particles L^−1^) [59,60], may negatively influence microbial dechlorination. This finding is in agreement with a recent study demonstrating a similar inhibitory effect of polyethylene, polypropylene and PS microplastics on the reductive dechlorination of PCBs [12], and the inhibition was attributed to a negative effect of MPs on microbial synergistic interactions in the culture system.

The inhibition of reductive dechlorination may vary depending on the particle size, where NP-sized plastic particles may enter microbial cells more easily through the membrane and influence microbial metabolic activity, inducing cell membrane disruption or the clogging of pores and channels essential for nutrient uptake and waste expulsion or oxidative stress [61]. In addition, smaller MP particles (10–50 µm) have a greater sorption capacity for organic pollutants, producing MP–pollutant clusters that are less bioavailable for microbial reduction and metabolism, causing an inhibition in degradation by weakening microbial synergistic interactions in the dechlorinating microbial community and effectively inducing a *cis*-DCE stall, when chlorinated solvents are present in an aquifer. PA6 microplastics, with their unique surface properties, can adsorb various chemical compounds from the environment, including essential nutrients or even inhibitory substances. This adsorption could deplete vital resources available to *Dehalococcoides* or concentrate toxic compounds on the microplastic surface, creating a localized environment detrimental to their activity. Exposure to MPs/NPs can cause growth inhibition and reactive oxygen species (ROS) generation, leading to oxidative stress [22,62]. The presence of NPs/MPs may disrupt cellular functions such as RDase activity and/or the flow of electrons and respiratory energy conservation. The inhibition is likely a combination of these physical, chemical and cellular disturbances rather than a single isolated mechanism.

### 3.3. Presence of Plastic Particles Has No Effect on RDX Degradation

**Figure 5:**
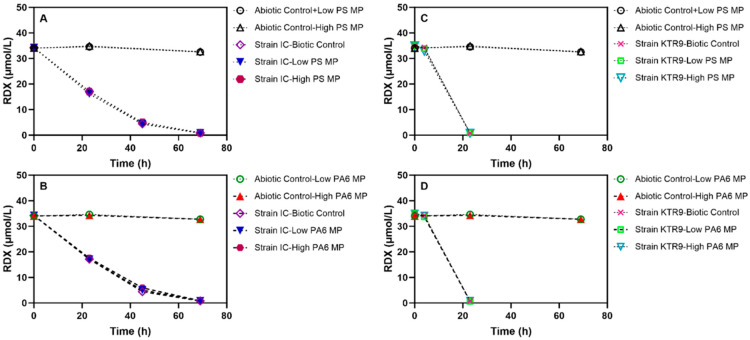
Effects of plastic presence on RDX degradation by *P. fluorescence* strain IC and *Gordonia* sp. strain KTR9. Low: 5.0 × 10^3^ particles mL^−1^; high: 5.0 × 10^5^ particles mL^−1^. PS: polystyrene, PA6: nylon 6. (**A**) RDX degradation by strain IC in the presence of 5.0 × 10^3^ and 5.0 × 10^5^ particles mL^−1^ PS MPs; (**B**) RDX degradation by strain IC in the presence of 5.0 × 10^3^ and 5.0 × 10^5^ particles mL^−1^ PA6 MPs; (**C**) RDX degradation by strain KTR9 in the presence of 5.0 × 10^3^ and 5.0 × 10^5^ particles mL^−1^ PS MPs; (**D**) RDX degradation by strain KTR9 in the presence of 5.0 × 10^3^ and 5.0 × 10^5^ particles mL^−1^ PA6 MPs. Vertical error bars denote the standard deviation.

The degradation of RDX by two pure cultures was evaluated in the presence of PS and PA6 MPs and NPs (Figure 5). Relative to the controls without plastics, the addition of a low or high concentration of plastic particles did not result in any change in the kinetics or extent of RDX degradation. The microbial growth and aerobic degradation of RDX by KTR9 was consistent with that reported previously in the literature [63], with a complete utilization of RDX in approximately 20 h.

As strain I-C degrades RDX anoxically, the degradation kinetics are slower in comparison to those observed in strain KTR9 cultures [39]. Hence, the observed depletion of the RDX took ~72 h. Additionally, the size of particles did not show any significant effect on RDX degradation. This result indicates that the presence of the PS and PA6 polymers had no effect on degradation of RDX. Thus, no further molecular biology analyses (qPCR, proteomics) were performed on these culture sets.

The use of pure cultures of strains IC and KTR9 for RDX to study the effects of PS and PA6 MPs and NPs on RDX degradation is a valuable approach for fundamental understanding. However, this approach does not account for the intricate dynamics inherent to natural microbial communities. Environmental consortia are highly diverse, featuring complex interspecies relationships (synergistic, antagonistic or competitive) that can profoundly alter their collective response to stressors like MPs and NPs. These interactions might buffer adverse effects or, alternatively, intensify them via indirect pathways such as altered nutrient cycling or the release of inhibitory metabolites by other species. Consequently, the inhibitory effects, or lack thereof, observed in a pure culture may not accurately predict the behavior of a mixed microbial community in a real-world bioremediation setting.

### 3.4. Effects of Plastic Particles on Dhc Abundance

**Figure 6:**
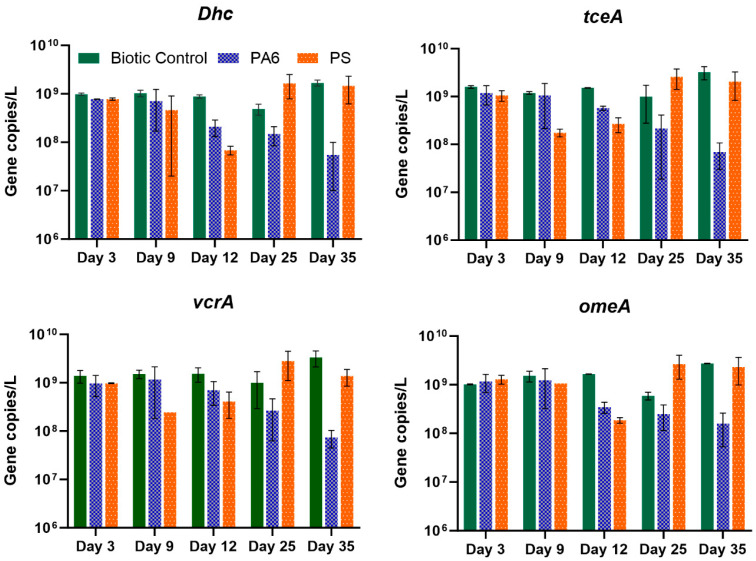
Abundance of reductive dehalogenase genes (*tceA*, *vcrA*) and *Dhc* and *omeA* genes in Set 1 samples in the presence PA6 and PS MPs. Each measurement represents an average of triplicate replicates. Error bars are one standard deviation.

The results depicted in Figure 6 demonstrate the impact of PA6 and PS MPs on *Dhc* cell abundance, as determined by the qPCR assay specific to *Dhc* 16S rRNA genes. The total abundance of *Dhc* cells in the biotic control (no particles added) remained consistent across the 35 days of the experimental time during the reductive dechlorination of TCE to *cis*-DCE, VC and ethene. The 9.79 × 10^8^ ± 5.46 × 10^7^ gene copies L^−1^ reported (Day 3) at the beginning of the experiment increased slightly by Day 35 to 1.68 × 10^9^ ± 2.41 × 10^8^ gene copies L^−1^. However, this variation is not statistically significant (*p* > 0.05). It is known that some *Dhc* strains drive the reductive dechlorination of PCE/TCE to VC and to ethene [3,64]. Microorganisms such as *Desulfitobacterium* can also dechlorinate PC and/or TCE to *cis-*DCE and are present in the SDC-9 consortium. However, *Dhc* strains are the only microorganisms within the SDC-9 consortium with proven *cis-*DCE- and VC-to-ethene dechlorination activity [43,65]. As evident by our prior studies that define SDC-9 microbial composition, the qPCR assay designed to detect *Dhc* abundance is the most direct method to verify any changes related to its performance [50].

When the SDC-9 culture was cultivated in the presence of PS particles, the total *Dhc* abundance decreased from 7.8 × 10^8^ ± 4.3 × 10^7^ on Day 3 to 6.9 × 10^7^ ± 1.4 × 10^7^ gene copies L^−1^ on Day 12. The abundance of the TCE-RDase (*tceA*) gene showed a similar trend, with a Day 3 *tceA* abundance of 1.1 × 10^9^ ± 2.6 × 10^8^ gene copies L^−1^ decreasing to 2.7 × 10^8^ ± 9.7 × 10^7^ gene copies L^−1^ on Day 12. The abundance of the VC RDase gene (*vcrA*) showed a declining trend from a Day 3 abundance of 9.7 × 10^8^ ± 1.8 × 10^7^ gene copies L^−1^ to 4.1 × 10^8^ ± 2.3 × 10^8^ gene copies L^−1^ on Day 12. Interestingly, the abundance of *Dhc* cells and target RDase genes increased to nearly 1.4–1.6 × 10^9^ gene copies L^−1^ by Day 25 and Day 35. This may be explained by biofilm formation. Saygin and Baysal et al. [66] reported that the biofilm formation of *E. coli* cells increased with higher concentrations of micro/submicron-sized plastic particles. The formation of biofilms over time apparently enabled the bacteria to withstand external stresses induced by the PS MPs, possibly resulting in reduced generation of reactive oxygen species (ROS) [26].

The most significant decrease in reductive dehalogenase gene abundance was observed when PA6 MPs were added to the SDC-9 consortium. On Day 12, *Dhc* cell number decreased from 7.7 × 10^8^ ± 8.6 × 10^6^ gene copies L^−1^ (Day 3) to 2.1 × 10^8^ ± 5.5 × 10^7^ gene copies L^−1^ (Day 12). With time, *Dhc* cell abundance continued to decrease consistently with the cessation of reductive dechlorination and the persistence of *cis*-DCE in the incubation vessels. Only 5.5 × 10^7^ ± 4.5 × 10^7^ gene copies L^−1^ were measured on Day 35 of the experiment. In parallel to the loss of the *Dhc* cells, the gene copies L^−1^ of *tceA* and *vcrA* decreased from 1.2 × 10^9^ ± 5.1 × 10^8^ (Day 3) to 6.8 × 10^7^ ± 3.8 × 10^7^ (Day 35) and 9.6 × 10^8^ ± 4.5 × 10^8^ (Day 3) to 7.4 × 10^7^ ± 2.9 × 10^7^ (Day 35), respectively. These results are in agreement with previous reports on conditions defined as a “*cis*-DCE stall” [67]. Observations on the presence of *Dehalococcoides* and the stalling of PCE/TCE dechlorination at *cis*-DCE or VC have been reported in soil and sediment microcosm studies and bench-scale bioremediation scenarios [67], as well as at contaminated sites undergoing bioremediation [49,68]. Whereas some of the abovementioned work did not put forth an explanation for the inability to achieve dechlorination of *cis-*DCE or VC, the absence of *Dehalococcoides mccartyi* strains with DCE- and VC-respiring metabolic capabilities may be a factor. However, in this study, the *cis-*DCE stall was directly related to the presence of PA6 particles and their effect on the reductive dechlorination of CVOCs. This effect can be explained by the reduction in *vcrA* gene abundance over time.

The concentrations of MPs/NPs employed in this study (e.g., 5 × 10^5^ particles/mL) were intentionally higher than typical average environmental concentrations. This approach was adopted to understand clear concentration–response relationships and identify potential mechanisms within controlled experimental timescales, especially given the analytical challenges in quantifying MPs/NPs at true environmental levels. However, it is likely that the effects observed at these elevated concentrations may be more pronounced than those occurring under the chronic, lower-dose exposures typical of many natural environments.

Current bioremediation monitoring regimes for CVOCs rely on *Dhc* nucleic acid biomarkers and inform about the potential for reductive dechlorination reactions but not the actual activity [43,49]. On the other hand, proteome analysis can detect and quantify proteins of interest (e.g., RDases catalyzing reductive dechlorination reactions) and generate information about functional (i.e., actual) activity. To this end, our team has developed a pipeline of proteomic tools that allow for the absolute quantification of RDases in environmental matrices [43]. These methods provide direct proof based on the expression of enzymes related to the dechlorination of chlorinated ethenes and allow for pinpointing metabolic shortcomings of the system that remains under evaluation.

**Figure 7:**
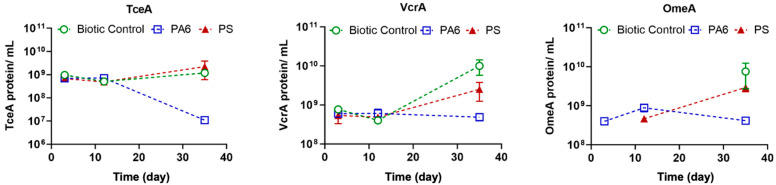
Proteomic data showing the abundance of TceA, VcrA and OmeA proteins in Set 1 samples in the presence of PA6 and PS MPs. Each data point represents triplicate measurement. Error bars are one standard deviation.

In this study, existing proteomics methods [43,69] were applied to examine whether the presence of PA6 and PS MPs had any inhibitory effect on the expression of enzymes directly responsible for dechlorination reactions. RDases are key respiratory enzymes involved in the anaerobic reductive dechlorination of chlorinated compounds, and TceA, VcrA and OmeA were reported among the most frequently identified proteins in dechlorinating culture [43,58,70]. The proteomic data are shown in Figure 7. The positive control shows protein expression patterns of TceA, VcrA and OmeA comparable to the data previously reported for the SDC-9 consortium [43,51]. The degradation of TCE to *cis*-DCE and the further formation of VC and ethene was correlated with an increased expression of reductive dehalogenases, namely TceA and VcrA [51]. However, when PA6 MPs were present in the culture vessels, the expression of TceA peptides encoding for the TCE reductive dehalogenase decreased from 6.9 × 10^8^ ± 9.6 × 10^7^ peptides mL^−1^ to 1.1 × 10^7^ ± 2.6 × 10^6^ peptides mL^−1^ in just 15 days. This decrease was correlated with the decrease in TCE degradation and persistence of *cis*-DCE in PA6-spiked microcosms. Moreover, additional proteomic data collected for the expression of the VC RDase showed a similar decreasing trend (5.9 × 10^8^ ± 1.0 × 10^8^ peptides mL^−1^ to 1.1 × 10^7^ ± 1.8 × 10^6^ peptides mL^−1^) related to persistence of cis-DCE and the decrease in the formation of VC and ethene, indicating a *cis*-DCE stall. These data are consistent with the CVOC results, demonstrating that despite the presence of *Dhc* cells, the formation of chlorinated TCE transformation products is significantly impacted by the presence of PA6 microparticles.

Hydrophobic polymers like polypropylene, polystyrene and polyethylene as hydrophobic pollutants and polymers with specific polar groups (like PA6) as polar contaminants are particularly concerning due to their strong sorption capabilities at bioremediation sites. Polymers like PA6, with their amide groups, can exhibit strong sorption for polar and semi-polar organic pollutants through hydrogen bonding and electrostatic interactions, as highlighted by the TCE sorption data.

## 4. Conclusions

Despite the increasing interest in the assessment of MP and NP occurrence in the environment, only a few reports currently present data on their effects on the biodegradation of groundwater contaminants [71]. Thus, the aim of this study was to evaluate the potential impacts of MPs and NPs of PS and PA6, the most commonly occurring groundwater plastics, on the degradation of selected explosive compounds and chlorinated ethenes. Specifically, organohalide-respiring bacteria present in the SDC-9 consortium and pure cultures of RDX degraders—*Gordonia* sp. KTR9 and *Pseudomonas fluorescens* I-C—were used to examine the effects of plastic particle size and type on microbial degradation. The sorption and degradation experiments conducted in this study highlight the complex interactions between micro/nanoplastics and CVOCs/RDX. While RDX showed negligible affinity for both micro- and nanoplastics, and cis-DCE, VC and ethene exhibited no significant sorption to PA6 and PS particles, TCE demonstrated notable sorption to PA6 microplastics. This differential sorption, particularly the higher affinity of TCE for PA6 microplastics than for other chlorinated compounds and PS particles, underscores the importance of polymer type, particle size and contaminant hydrophobicity in abiotic interactions. Importantly, the observed sorption of TCE to PA6 microplastics suggests a potential mechanism by which these particles may decrease the aqueous concentration of organic pollutants, thereby influencing their environmental fate and bioavailability for microbial degradation.

Beyond abiotic interactions, our findings reveal a significant inhibitory effect of high concentrations of PA6 microplastics on the reductive dechlorination of TCE by the SDC-9 microbial consortium, leading to a “*cis*-DCE stall” and a dramatic reduction in the abundance and activity of *Dehalococcoides* cells and key reductive dehalogenase enzymes (TceA and VcrA). In contrast, PS microplastics and both PA6 and PS nanoplastics showed no comparable inhibitory effects on CVOCs degradation, nor did either plastic type impact RDX degradation.

RDases such as TceA and VcrA, as well as the electron shuttling protein OmeA, implicated in cVOC degradation occur in the periplasm, in contrast to RDX degradation proteins that are of cytoplasmic localization. TCE-RDase is a peripheral membrane-bound protein of the cytoplasmic membrane that serves as one of two identified terminal reductases in the electron transport chain of reductive dechlorinators such as *Dhc* [71]. TCE-RDase accepts electrons from an unknown electron donor or donors (probably located in the membrane), which in turn receive electrons from a membrane-bound hydrogenase. Additional reports for the *Dhc* strain 195 confirmed that the RDases in this organism have an exoplasmic localization [72]. It is possible that PA6 MPs are transported and accumulate in higher concentrations than PS or even PA6 NPs, affecting RDase turnover. The PA6 MPs are also of different morphology, with a fiber-like shape, in contrast to the more spherical particles of PS. This difference in shape and size may contribute to overall *Dhc* cell performance upon exposure. Additional studies will have to be performed to fully understand this phenomenon. However, these results are of critical importance to the future planning of remediation activities in groundwaters that show a presence of PA6 particles. Several studies show that positively charged MPs bind cells because of electrostatic attraction, while other negatively charged MPs bind cells loosely through van der Waals forces and acid–base interactions, together with electrostatic force. In other words, positively charged microplastics may cause toxic effects or stress on cells due to electrostatic attraction, while negatively charged microplastics are mainly aggregated. As demonstrated in this study, MPs affect the activity of some bacterial strains like *Dhc* cells and may inhibit the degradation of chlorinated ethenes and other detoxification processes of importance to bioremediation cleanup goals.

The results collectively indicate that the presence of certain microplastic types at environmentally relevant high concentrations can detrimentally affect crucial microbial bioremediation processes for chlorinated volatile organic compounds. This has critical implications for understanding contaminant mobility, bioavailability and the efficacy of natural attenuation or engineered bioremediation strategies in environments impacted by both legacy pollutants and emerging plastic contaminants.

## Data Availability

The data presented in this study are available on request from the corresponding author.

## Supplementary Materials

The following supporting information can be downloaded at: https://www.mdpi.com/article/10.3390/toxics13080656/s1, Table S1: Primers and probes used in this study; Figure S1: Determination of average particle size distribution of PA6 (Nylon 6) sample using dynamic light scattering; Figure S2: Adsorption behavior of RDX in the presence of 5 × 10^3^ and 5 × 10^5^ PS and PA6 microplastics particles per mL. Each time point is plotted as an average of duplicate measurements. Error bars are calculated as the standard error from duplicate measurements; Figure S3: CVOC and ethene data for Set 2. Effect of 5.0 × 10^5^ particles per mL of MPs on reductive dechlorination of TCE. (**A**) Biotic control MP, SDC-9 dechlorinating culture without any plastic addition; (**B**) addition of 5 × 10^5^ particles PA6 (20 µm) on Day 3.

## Abbreviations

MPs, Microplastics; NPs, Nanoplastics; CVOCs, Chlorinated volatile organic contaminants; PCE, Tetrachloroethene; RDX, Hexahydro-1,3,5-trinitro-1,3,5-triazine; PS, Polystyrene; PA6, Polyamide 6; SDC-9, CVOC-degrading commercially available community; TCE, Trichloroethene; cis-DCE, cis-1,2-dichloroethene; VC, Vinyl chloride; *Dhc*, *Dehalococcoides mccartyi*; PAH, Polycyclic aromatic hydrocarbons; PCBs, Polycyclic chlorinated biphenyls; nifH, Nitrogenase reductase; amoA, Ammonium monooxygenase; BSM, Basal salt medium; BSM-N, Basal salt medium without ammonium; HPLC, High-pressure liquid chromatography; FID, Flame ionization detector; GC, Gas chromatograph; iRT, Indexed retention time; RDase, Reductive dehalogenase; OmeA, Organohalide respiration molybdoenzyme; TceA, TCE to VC RDase; VcrA, cis-DCE/VC-to-ethene RDase; *tceA*, Gene encoding TceA RDase; *vcrA*, Gene encoding VcrA RDase; qPCR, Quantitative PCR.

## References

[1] Li P., Karunanidhi D., Subramani T., Srinivasamoorthy K. (2021). Sources and consequences of groundwater contamination. Arch. Environ. Contam. Toxicol..

[2] Young T.S., Morley M.C., Snow D.D. (2006). Anaerobic biodegradation of RDX and TCE: Single-and dual-contaminant batch tests. Pract. Period. Hazard. Toxic Radioact. Waste Manag..

[3] He J., Sung Y., Krajmalnik-Brown R., Ritalahti K.M., Löffler F.E. (2005). Isolation and characterization of Dehalococcoides sp. strain FL2, a trichloroethene (TCE)-and 1,2-dichloroethene-respiring anaerobe. Environ. Microbiol..

[4] Krajmalnik-Brown R., Hölscher T., Thomson I.N., Saunders F.M., Ritalahti K.M., Löffler F.E. (2004). Genetic Identification of a Putative Vinyl Chloride Reductase in *Dehalococcoides* sp. Strain BAV1. Appl. Environ. Microbiol..

[5] Chen S., Chin Y., Yang H., Lu C., Liu M. (2021). Cometabolic biodegradation of chlorinated ethenes with methanotrophs in anaerobic/aerobic simulated aquifer. J. Environ. Biol..

[6] Fuller M.E., Hatzinger P.B., Condee C.W., Andaya C., Vainberg S., Michalsen M.M., Crocker F.H., Indest K.J., Jung C.M., Eaton H. (2015). Laboratory evaluation of bioaugmentation for aerobic treatment of RDX in groundwater. Biodegradation.

[7] Fournier D., Halasz A., Spain J., Spanggord R.J., Bottaro J.C., Hawari J. (2004). Biodegradation of the hexahydro-1, 3, 5-trinitro-1, 3, 5-triazine ring cleavage product 4-nitro-2, 4-diazabutanal by Phanerochaete chrysosporium. Appl. Environ. Microbiol..

[8] Halasz A., Hawari J. (2011). Degradation routes of RDX in various redox systems. Aquatic Redox Chemistry.

[9] Halasz A., Spain J., Paquet L., Beaulieu C., Hawari J. (2002). Insights into the formation and degradation mechanisms of methylenedinitramine during the incubation of RDX with anaerobic sludge. Environ. Sci. Technol..

[10] Rylott E.L., Jackson R.G., Edwards J., Womack G.L., Seth-Smith H.M.B., Rathbone D.A., Strand S.E., Bruce N.C. (2006). An explosive-degrading cytochrome P450 activity and its targeted application for the phytoremediation of RDX. Nat. Biotechnol..

[11] Fuller M.E., McClay K., Hawari J., Paquet L., Malone T.E., Fox B.G., Steffan R.J. (2009). Transformation of RDX and other energetic compounds by xenobiotic reductases XenA and XenB. Appl. Microbiol. Biotechnol..

[12] Li X., Xu Q., Cheng Y., Chen C., Shen C., Zhang C., Zheng D., Zhang D. (2022). Effect of microplastics on microbial dechlorination of a polychlorinated biphenyl mixture (Aroclor 1260). Sci. Total Environ..

[13] Liu J., Xu G., Zhao S., Chen C., Rogers M.J., He J. (2023). Mechanistic and microbial ecological insights into the impacts of micro-and nano-plastics on microbial reductive dehalogenation of organohalide pollutants. J. Hazard. Mater..

[14] Rosato A., Barone M., Negroni A., Brigidi P., Fava F., Xu P., Candela M., Zanaroli G. (2020). Microbial colonization of different microplastic types and biotransformation of sorbed PCBs by a marine anaerobic bacterial community. Sci. Total Environ..

[15] Basumatary T., Biswas D., Boro S., Nava A.R., Narayan M., Sarma H. (2025). Dynamics and Impacts of Microplastics (MPs) and Nanoplastics (NPs) on Ecosystems and Biogeochemical Processes: The Need for Robust Regulatory Frameworks. ACS Omega.

[16] Aralappanavar V.K., Mukhopadhyay R., Yu Y., Liu J., Bhatnagar A., Praveena S.M., Li Y., Paller M., Adyel T.M., Rinklebe J. (2024). Effects of microplastics on soil microorganisms and microbial functions in nutrients and carbon cycling—A review. Sci. Total Environ..

[17] Cole M., Lindeque P., Halsband C., Galloway T.S. (2011). Microplastics as contaminants in the marine environment: A review. Mar. Pollut. Bull..

[18] Reynaud S., Aynard A., Grassl B., Gigault J. (2022). Nanoplastics: From model materials to colloidal fate. Curr. Opin. Colloid Interface Sci..

[19] Fries E., Zarfl C. (2012). Sorption of polycyclic aromatic hydrocarbons (PAHs) to low and high density polyethylene (PE). Environ. Sci. Pollut. Res..

[20] Näkki P., Eronen-Rasimus E., Kaartokallio H., Kankaanpää H., Setälä O., Vahtera E., Lehtiniemi M. (2021). Polycyclic aromatic hydrocarbon sorption and bacterial community composition of biodegradable and conventional plastics incubated in coastal sediments. Sci. Total Environ..

[21] Rochman C.M., Hoh E., Hentschel B.T., Kaye S. (2013). Long-term field measurement of sorption of organic contaminants to five types of plastic pellets: Implications for plastic marine debris. Environ. Sci. Technol..

[22] Liu Q., Li L., Zhao X., Song K. (2021). An evaluation of the effects of nanoplastics on the removal of activated-sludge nutrients and production of short chain fatty acid. Process Saf. Environ. Prot..

[23] Yang X., He Q., Guo F., Sun X., Zhang J., Chen M., Vymazal J., Chen Y. (2020). Nanoplastics disturb nitrogen removal in constructed wetlands: Responses of microbes and macrophytes. Environ. Sci. Technol..

[24] Wei W., Huang Q.-S., Sun J., Dai X., Ni B.-J. (2019). Revealing the mechanisms of polyethylene microplastics affecting anaerobic digestion of waste activated sludge. Environ. Sci. Technol..

[25] Rong L., Zhao L., Zhao L., Cheng Z., Yao Y., Yuan C., Wang L., Sun H. (2021). LDPE microplastics affect soil microbial communities and nitrogen cycling. Sci. Total Environ..

[26] Kim S.Y., Kim Y.J., Lee S.-W., Lee E.-H. (2022). Interactions between bacteria and nano (micro)-sized polystyrene particles by bacterial responses and microscopy. Chemosphere.

[27] Nath J., De J., Sur S., Banerjee P. (2023). Interaction of Microbes with Microplastics and Nanoplastics in the Agroecosystems—Impact on Antimicrobial Resistance. Pathogens.

[28] Wang R., Li X., Li J., Dai W., Luan Y. (2023). Bacterial Interactions with Nanoplastics and the Environmental Effects They Cause. Fermentation.

[29] Sun X., Chen B., Li Q., Liu N., Xia B., Zhu L., Qu K. (2018). Toxicities of polystyrene nano-and microplastics toward marine bacterium Halomonas alkaliphila. Sci. Total Environ..

[30] Okshevsky M., Gautier E., Farner J.M., Schreiber L., Tufenkji N. (2020). Biofilm formation by marine bacteria is impacted by concentration and surface functionalization of polystyrene nanoparticles in a species-specific manner. Environ. Microbiol. Rep..

[31] Gao Z., Qian H., Cui T., Ren Z., Wang X. (2024). Comprehensive meta-analysis reveals the impact of non-biodegradable plastic pollution on methane production in anaerobic digestion. Chem. Eng. J..

[32] Azizi S.M.M., Hai F.I., Lu W., Al-Mamun A., Dhar B.R. (2021). A review of mechanisms underlying the impacts of (nano) microplastics on anaerobic digestion. Bioresour. Technol..

[33] Liu J., Xu G., Zhao S., He J. (2023). Resilience and functional redundancy of methanogenic digestion microbiome safeguard recovery of methanogenesis activity under the stress induced by microplastics. Mlife.

[34] Busch P.L., Stumm W. (1968). Chemical interactions in the aggregation of bacteria bioflocculation in waste treatment. Environ. Sci. Technol..

[35] Ning Q., Wang D., An J., Ding Q., Huang Z., Zou Y., Wu F., You J. (2022). Combined effects of nanosized polystyrene and erythromycin on bacterial growth and resistance mutations in Escherichia coli. J. Hazard. Mater..

[36] Harris P.T. (2020). The fate of microplastic in marine sedimentary environments: A review and synthesis. Mar. Pollut. Bull..

[37] Mammo F., Amoah I., Gani K., Pillay L., Ratha S., Bux F., Kumari S. (2020). Microplastics in the environment: Interactions with microbes and chemical contaminants. Sci. Total Environ..

[38] Crespy D., Landfester K. (2007). Preparation of nylon 6 nanoparticles and nanocapsules by two novel miniemulsion/solvent displacement hybrid techniques. Macromol. Chem. Phys..

[39] Ren Z., Gui X., Xu X., Zhao L., Qiu H., Cao X. (2021). Microplastics in the soil-groundwater environment: Aging, migration, and co-transport of contaminants–a critical review. J. Hazard. Mater..

[40] Zheng X., Chen Y., Wu R. (2011). Long-term effects of titanium dioxide nanoparticles on nitrogen and phosphorus removal from wastewater and bacterial community shift in activated sludge. Environ. Sci. Technol..

[41] Vainberg S., Mcclay K., Schaefer C., Steffan R. Dechlorination of mixed chlorinated solvents by a commercially available culture. Proceedings of the 9th International In Situ and On-Site Bioremediation Symposium.

[42] Schaefer C.E., Condee C.W., Vainberg S., Steffan R.J. (2009). Bioaugmentation for chlorinated ethenes using Dehalococcoides sp.: Comparison between batch and column experiments. Chemosphere.

[43] Kucharzyk K.H., Meisel J.E., Kara-Murdoch F., Murdoch R.W., Higgins S.A., Vainberg S., Bartling C.M., Mullins L., Hatzinger P.B., Löffler F.E. (2020). Metagenome-Guided Proteomic Quantification of Reductive Dehalogenases in the Dehalococcoides mccartyi-Containing Consortium SDC-9. J. Proteome Res..

[44] Ritalahti K.M., Amos B.K., Sung Y., Wu Q., Koenigsberg S.S., Löffler F.E. (2006). Quantitative PCR targeting 16S rRNA and reductive dehalogenase genes simultaneously monitors multiple Dehalococcoides strains. Appl. Environ. Microbiol..

[45] Thompson K.T., Crocker F.H., Fredrickson H.L. (2005). Mineralization of the cyclic nitramine explosive hexahydro-1, 3, 5-trinitro-1, 3, 5-triazine by *Gordonia* and *Williamsia* spp.. Appl. Environ. Microbiol..

[46] Hareland W.A., Crawford R.L., Chapman P.J., Dagley S. (1975). Metabolic function and properties of 4-hydroxyphenylacetic acid 1-hydroxylase from Pseudomonas acidovorans. J. Bacteriol..

[47] Yang Y., Cápiro N.L., Marcet T.F., Yan J., Pennell K.D., Löffler F.E. (2017). Organohalide respiration with chlorinated ethenes under low pH conditions. Environ. Sci. Technol..

[48] Fuller M.E., Farquharson E.M., Hedman P.C., Chiu P. (2022). Removal of munition constituents in stormwater runoff: Screening of native and cationized cellulosic sorbents for removal of insensitive munition constituents NTO, DNAN, and NQ, and legacy munition constituents HMX, RDX, TNT, and perchlorate. J. Hazard. Mater..

[49] Michalsen M.M., Kara Murdoch F., Löffler F.E., Wilson J., Hatzinger P.B., Istok J.D., Mullins L., Hill A., Murdoch R.W., Condee C. (2021). Quantitative Proteomics and Quantitative PCR as Predictors of cis-1, 2-Dichlorethene and Vinyl Chloride Reductive Dechlorination Rates in Bioaugmented Aquifer Microcosms. ACS EST Eng..

[50] Michalsen M., Kucharzyk K., Bartling C., Meisel J., Hatzinger P., Wilson J., Solutions S.E., Istok L.J., Murdoch F.K., Löffler F. (2020). Validation of Advanced Molecular Biological Tools to Monitor Chlorinated Solvent Bioremediation and Estimate cVOC Degradation Rates.

[51] Avila M.A.S., Breiter R. (2008). Competitive sorption of cis-DCE and TCE in silica gel as a model porous mineral solid. Chemosphere.

[52] Fu L., Li J., Wang G., Luan Y., Dai W. (2021). Adsorption behavior of organic pollutants on microplastics. Ecotoxicol. Environ. Saf..

[53] Rai P.K., Sonne C., Brown R.J., Younis S.A., Kim K.-H. (2022). Adsorption of environmental contaminants on micro-and nano-scale plastic polymers and the influence of weathering processes on their adsorptive attributes. J. Hazard. Mater..

[54] Brusseau M.L., Rao P.S.C., Gillham R.W. (1989). Sorption nonideality during organic contaminant transport in porous media. Crit. Rev. Environ. Control..

[55] Velzeboer I., Kwadijk C.J.A.F., Koelmans A.A. (2014). Strong Sorption of PCBs to Nanoplastics, Microplastics, Carbon Nanotubes, and Fullerenes. Environ. Sci. Technol..

[56] Yu S., Dolan M.E., Semprini L. (2005). Kinetics and Inhibition of Reductive Dechlorination of Chlorinated Ethylenes by Two Different Mixed Cultures. Environ. Sci. Technol..

[57] Loffler F.E., Ritalahti K.M., Tiedje J.M. (1997). Dechlorination of chloroethenes is inhibited by 2-bromoethanesulfonate in the absence of methanogens. Appl. Environ. Microbiol..

[58] Natesan U., Vaikunth R., Kumar P., Ruthra R., Srinivasalu S. (2021). Spatial distribution of microplastic concentration around landfill sites and its potential risk on groundwater. Chemosphere.

[59] Koelmans A.A., Mohamed Nor N.H., Hermsen E., Kooi M., Mintenig S.M., De France J. (2019). Microplastics in freshwaters and drinking water: Critical review and assessment of data quality. Water Res..

[60] Matthews S., Mai L., Jeong C.-B., Lee J.-S., Zeng E.Y., Xu E.G. (2021). Key mechanisms of micro-and nanoplastic (MNP) toxicity across taxonomic groups. Comp. Biochem. Physiol. Part C Toxicol. Pharmacol..

[61] Yang J.-Q., Li Z.-L., Wu B., Jin Y.-R., Cao D., Nan J., Chen X.-Q., Liu W.-Z., Gao S.-H., Wang A.-J. (2023). Insights into the influence on 2, 4, 6-trichlorophenol microbial reductive dechlorination process by exposure to microplastics. J. Hazard. Mater..

[62] Zhu S.-H., Reuther J., Liu J., Crocker F.H., Indest K.J., Eltis L.D., Mohn W.W. (2015). The essential role of nitrogen limitation in expression of xplA and degradation of hexahydro-1,3,5-trinitro-1,3,5-triazine (RDX) in Gordonia sp. strain KTR9. Appl. Microbiol. Biotechnol..

[63] Hendrickson E.R., Payne J.A., Young R.M., Starr M.G., Perry M.P., Fahnestock S., Ellis D.E., Ebersole R.C. (2002). Molecular analysis of Dehalococcoides 16S ribosomal DNA from chloroethene-contaminated sites throughout North America and Europe. Appl. Environ. Microbiol..

[64] Dang H., Kanitkar Y.H., Stedtfeld R.D., Hatzinger P.B., Hashsham S.A., Cupples A.M. (2018). Abundance of chlorinated solvent and 1, 4-dioxane degrading microorganisms at five chlorinated solvent contaminated sites determined via shotgun sequencing. Environ. Sci. Technol..

[65] Saygin H., Baysal A. (2020). Biofilm formation of clinically important bacteria on bio-based and conventional micro/submicron-sized plastics. Bull. Environ. Contam. Toxicol..

[66] Fennell D.E., Carroll A.B., Gossett J.M., Zinder S.H. (2001). Assessment of indigenous reductive dechlorinating potential at a TCE-contaminated site using microcosms, polymerase chain reaction analysis, and site data. Environ. Sci. Technol..

[67] Harkness M.R., Bracco A.A., Brennan M.J., DeWeerd K.A., Spivack J.L. (1999). Use of bioaugmentation to stimulate complete reductive dechlorination of trichloroethene in Dover soil columns. Environ. Sci. Technol..

[68] Duhamel M., Wehr S.D., Yu L., Rizvi H., Seepersad D., Dworatzek S., Cox E.E., Edwards E.A. (2002). Comparison of anaerobic dechlorinating enrichment cultures maintained on tetrachloroethene, trichloroethene, cis-dichloroethene and vinyl chloride. Water Res..

[69] Morris R., Fung J., Rahm B., Zhang S., Freedman D., Zinder S., Richardson R. (2007). Comparative proteomics of Dehalococcoides spp. reveals strain-specific peptides associated with activity. Appl. Environ. Microbiol..

[70] Yan J., Wang J., Villalobos Solis M.I., Jin H., Chourey K., Li X., Yang Y., Yin Y., Hettich R.L., Löffler F.E. (2021). Respiratory vinyl chloride reductive dechlorination to ethene in TceA-expressing Dehalococcoides mccartyi. Environ. Sci. Technol..

[71] Magnuson Jon K., Romine Margaret F., Burris David R., Kingsley Mark T. (2000). Trichloroethene Reductive Dehalogenase fromDehalococcoides ethenogenes: Sequence of tceA and Substrate Range Characterization. Appl. Environ. Microbiol..

[72] Nijenhuis I., Zinder Stephen H. (2005). Characterization of Hydrogenase and Reductive Dehalogenase Activities of Dehalococcoides ethenogenes Strain 195. Appl. Environ. Microbiol..

