# Supplementary material for: Presence of Micro- and Nanoplastics Affects Degradation of Chlorinated Solvents
Source: Toxics. 2025 Jul 31;13(8):656. doi: 10.3390/toxics13080656 (PMC12390433; doi:10.3390/toxics13080656)
Supplement: Supplementary file 1 [file toxics-13-00656-s001.zip › toxics-3719097-supplementary.pdf]

Supplementary Materials

Presence of Micro- and Nanoplastics Affects Degradation of Chlorinated Solvents

Fadime Kara Murdoch [1], Yanchen Sun [2,3,4], Mark E. Fuller [5], Larry Mullins [1], Amy Hill [1], Jacob Lilly [1], John Wilson [6], Frank E. Löffler [2,3,4,7] and Katarzyna H. Kucharzyk [1,*]

[1] Battelle Memorial Institute, 505 King Ave, Columbus, OH 43201, USA; karamurdoch@battelle.org (F.K.M.); mullinsl@battelle.org (L.M.); hilla@battelle.org (A.H.); lillyj@battelle.org (J.L.)
[2] Department of Civil and Environmental Engineering, University of Tennessee, Knoxville, TN 37996, USA; yanchen.sun@whoi.edu (Y.S.); frank.loeffler@utk.edu (F.E.L.)
[3] Department of Biochemistry & Cellular and Molecular Biology, University of Tennessee, Knoxville, TN 37996, USA
[4] Department of Biosystems Engineering, University of Tennessee, Knoxville, TN 37996, USA
[5] Aptim Federal Services, LLC, 17 Princess Road, Lawrenceville, NJ 08648, USA; mark.fuller@aptim.com
[6] Scissortail Environmental Solutions, LLC, 2013 Foster Drive, Ada, OK 74820, USA; john@scissortailenv.com
[7] Biosciences Division, Oak Ridge National Laboratory, Oak Ridge, TN 37831, USA
* Correspondence: kucharzyk@battelle.org; Tel.: +614-424-5489

Materials and Methods

Quantitative Proteomics (qProt). Mass spectrometric parameters for the analysis of target proteins on the Xevo TQ-XS triple quadrupole mass spectrometer were optimized for *Dhc* biomarker peptides. Isotopically labeled peptides were injected via syringe infusion, and precursor-to-product ion transitions were identified and optimized using the IntelliStart feature in MassLynx v4.2 software. The instrumentation was controlled using MassLynx v4.2 software. 5 µL of sample was injected onto a 0.3 mm x 50 mm Waters M-Class Symmetry C18 trapping column (5 µm particle size, 100 Å pore size) using a trap-and-elute method. Samples were washed for 5 minutes using 99:1 solvent A to B prior to gradient separation on the analytical column. Solvents used included 0.1% formic acid (v/v) in water (solvent A) and 0.1% formic acid (v/v) in acetonitrile (solvent B). Peptide separation was achieved using a 0.3 mm x 150 mm Waters M-Class HSS T3 analytical column (1.8 µm particle size, 100 Å pore size) and a linear gradient as follows: (1) 1% solvent B in A (from 0-5 min), (2) 1-35% solvent B in A (from 5-65 min), (3) 35-90% solvent B in A (from 65- 66 minutes), and (4) 90% solvent B in A (from 66-70 minutes), with a total runtime of 90 min, including mobile phase equilibration. Mass spectrometric analysis was performed using the SRM method previously described by Kucharzyk et al. Resulting data was processed using Skyline software (MacCoss Lab Software). Native peptide concentrations were determined by comparing the peak ratios of the most abundant precursor-to-product ion transitions of the native and isotopically labeled (heavy) peptides.

Table S1. Primers and probes used in this study.

Primer/probe	Primer/probe sequence (5'-3')	Target	Amplicon size (bp)	Reference
*Dhc*1200F	CTGGAGCTAATCCCCAAAGCT	*Dhc* 16S rRNA gene	66	[44]
*Dhc*1271R	CAACTTCATGCAGGCGGG			
*Dhc*1240Probe	FAM-TCGGATTGCAGGCTGA-MGB			
*tceA*1270F	ATCCAGATTATGACCCTGGTGAA	*tceA* gene	67	[49]
*tceA*1336R	GCGGCATATATTAGGGCATCTT			
*tceA*1294Probe	FAM-TGGGCTATGGCGACCGCAGG-MGB			
*vcrA*1022F	CGGGCGGATGCACTATTTT	*vcrA* gene	72	[44]
*vcrA*1093R	GAATAGTCCGTGCCCTTCCTC			
*vcrA*1046Probe*	FAM-TACCAGGAAATGGTTGAGTTAC-MGB			
*omeA*F	ATGGGTCCACGGAAAAGACC	*omeA* gene	62	[51]
*omeA*R	CTCAAGATCAGCGGAGGGAC			
*omeA*Probe	FAM-AGCTGTGGCAAAGAGACCC-MGB			

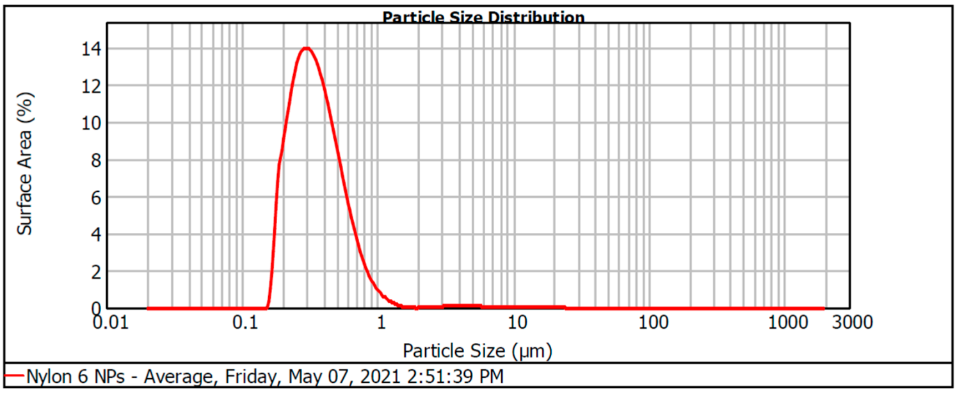

Figure S1. Determination of average particle size distribution of PA6 (Nylon 6) sample using dynamic light scattering technique.

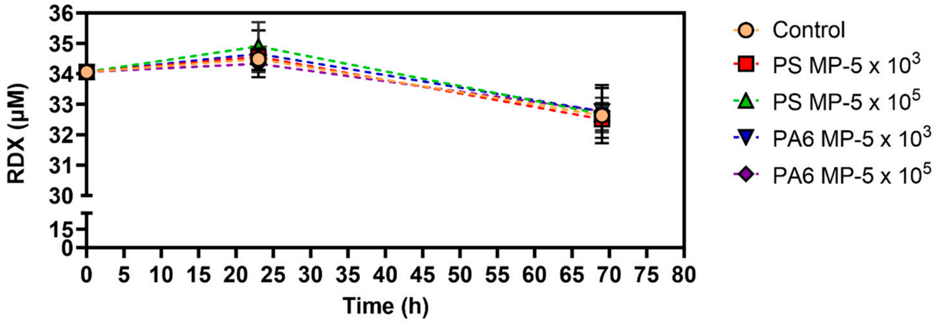

Figure S2. Adsorption behavior of RDX in the presence of 5 x 10^3 and 5 x 10^5 PS and PA6 microplastics particles per mL. Each time point is plotted as an average of duplicate. Error bars are calculated as a standard error on duplicate measurement.

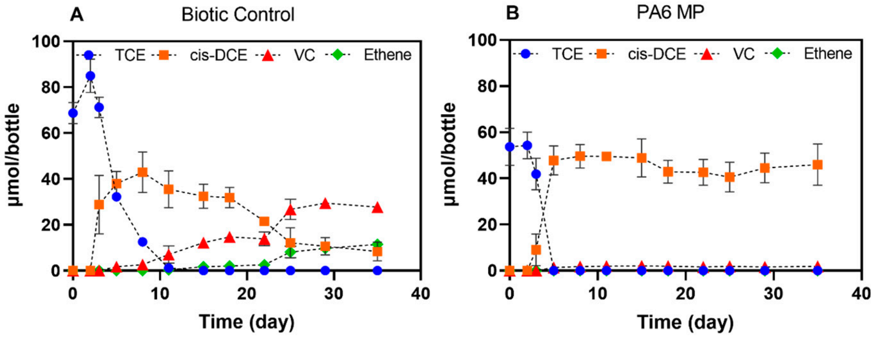

Figure S3. CVOCs and ethene data for Set 2. Effect of 5.0 x 105 particles per mL of MPs on reductive dechlorination of TCE. **(A)** Biotic Control MP, SDC-9 dechlorinating culture without any plastic addition; **(B)** addition of 5 x 10^5 particles PA6 (20 µm) on Day 3.
